# Crystal structure of 4,6-bis­[(*E*)-4-bromo­styr­yl]-2-(butyl­sulfan­yl)pyrimidine

**DOI:** 10.1107/S1600536814024714

**Published:** 2014-11-21

**Authors:** Wang Yu, Jingbao Song, Aijian Wang

**Affiliations:** a China–Australia Joint Research Center for Functional Molecular Materials, Scientific Research Academy & School of Chemistry and Chemical Engineering, Jiangsu University, Zhenjiang 212013, People’s Republic of China

**Keywords:** crystal structure, weak inter­action, pyrimidine

## Abstract

In the title compound, C_24_H_22_Br_2_N_2_S, the dihedral angles between the central pyrimidine ring and the pendant bromo­benzene rings are 11.02 (11) and 13.20 (12)°. The butyl side chain adopts a *gauche* conformation [C—C—C—C = −67.4 (4)°]. In the crystal, weak aromatic π–π stacking is observed between the pyrimidine ring and one of the benzene rings [centroid–centroid separation = 3.6718 (17) Å].

## Related literature   

For general background to pyrimidine derivatives and their applications, see: Walker *et al.* (2009 ▶); van Laar *et al.* (2001 ▶); Joule & Mills (2000 ▶); Deng *et al.* (2008 ▶); Nguyen (2008 ▶). For further synthetic details, see: Liu *et al.* (2007 ▶).
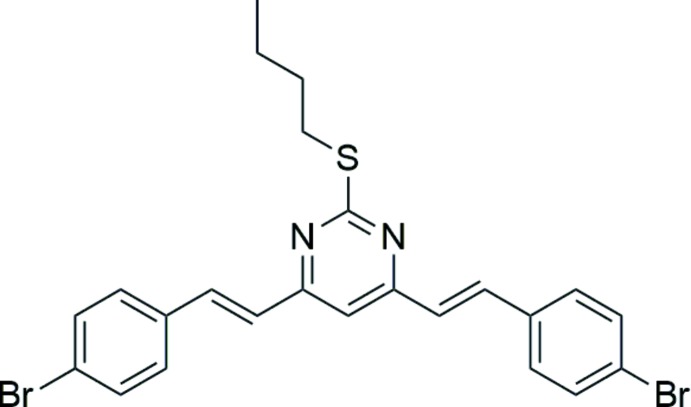


## Experimental   

### Crystal data  

C_24_H_22_Br_2_N_2_S
*M* *_r_* = 530.32
Monoclinic, 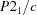

*a* = 9.5636 (19) Å
*b* = 10.630 (2) Å
*c* = 23.708 (6) Å
β = 112.82 (3)°
*V* = 2221.5 (10) Å^3^
*Z* = 4
Mo *K*α radiation
μ = 3.76 mm^−1^
*T* = 153 K
0.24 × 0.22 × 0.20 mm

### Data collection  

Rigaku Saturn724+ diffractometer
Absorption correction: multi-scan (*CrystalClear*; Rigaku, 2008 ▶) *T*_min_ = 0.728, *T*_max_ = 1.000
10036 measured reflections
4043 independent reflections
3601 reflections with *I* > 2σ(*I*)
*R*_int_ = 0.022

### Refinement  

*R*[*F* ^2^ > 2σ(*F* ^2^)] = 0.030
*wR*(*F* ^2^) = 0.066
*S* = 1.04
4043 reflections
263 parameters
H-atom parameters constrained
Δρ_max_ = 0.68 e Å^−3^
Δρ_min_ = −0.38 e Å^−3^

### 

Data collection: *CrystalClear* (Rigaku, 2008 ▶); cell refinement: *CrystalClear*; data reduction: *CrystalClear*; program(s) used to solve structure: *SHELXTL* (Sheldrick, 2008 ▶); program(s) used to refine structure: *SHELXTL*; molecular graphics: *SHELXTL*; software used to prepare material for publication: *SHELXTL*.

## Supplementary Material

Crystal structure: contains datablock(s) I, New_Global_Publ_Block. DOI: 10.1107/S1600536814024714/hb7313sup1.cif

Structure factors: contains datablock(s) I. DOI: 10.1107/S1600536814024714/hb7313Isup2.hkl

Supporting information file. DOI: 10.1107/S1600536814024714/hb7313Isup3.cml

Supporting information file. DOI: 10.1107/S1600536814024714/hb7313Isup4.doc

**Fig. 1.**
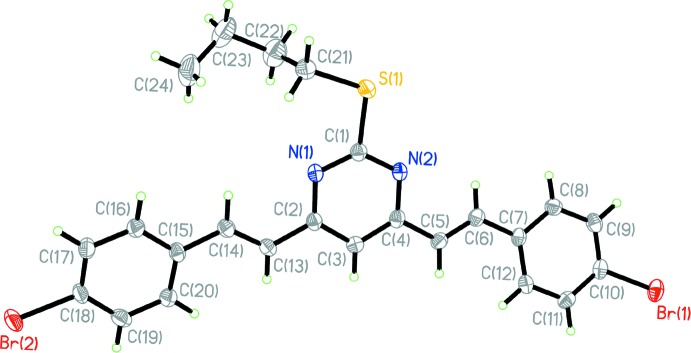
The molecular structure of (I) showing 50% displacement ellipsoids.

. DOI: 10.1107/S1600536814024714/hb7313fig1.tif
The mol­ecular structure of (I) showing 50% displacement ellipsoids.

**Fig. 2.**
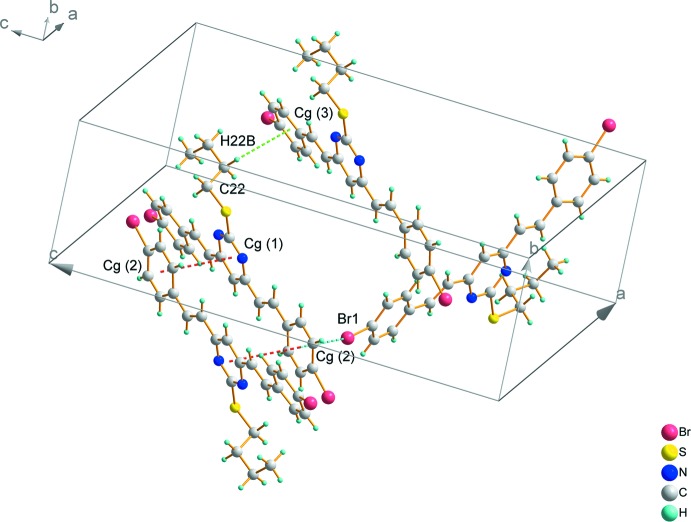
Packing diagram for (I)

. DOI: 10.1107/S1600536814024714/hb7313fig2.tif
Packing diagram for (I)

CCDC reference: 1010673

Additional supporting information:  crystallographic information; 3D view; checkCIF report

## supplementary crystallographic information

### S1.1. Synthesis and crystallization

2-(Butyl­thio)-4,6-di­methyl­pyrimidine (2.95 g, 15 mmol) and bromo­benzaldehyde (6.1 g, 33 mmol) were added in an aqueous solution of sodium hydroxide (5 M, 50 ml) containing tetra­butyl­ammonium iodide (10 mol % versus the heterocycle) and mixed. The mixture was heated under reflux for 3 h. After cooling, the reaction mixture was extracted with di­chloro­methane (120 ml × 3). The extract solution was dried with magnesium sulfate. After removal of the drying agent by filtration, the solvent was removed by evaporation under reduced pressure. The crude product was recrystallized to afford yellow prisms of the title compound, (I) (5.17 g, yield 65%).

### Crystal data

**Table:**

C_24_H_22_Br_2_N_2_S	*F*(000) = 1064
*M**_r_* = 530.32	*D*_x_ = 1.586 Mg m^−^^3^
Monoclinic, *P*2_1_/*c*	Mo *K*α radiation, λ = 0.71073 Å
*a* = 9.5636 (19) Å	Cell parameters from 9235 reflections
*b* = 10.630 (2) Å	θ = 2.9–29.2°
*c* = 23.708 (6) Å	µ = 3.76 mm^−^^1^
β = 112.82 (3)°	*T* = 153 K
*V* = 2221.5 (10) Å^3^	Prism, yellow
*Z* = 4	0.24 × 0.22 × 0.20 mm

### Data collection

**Table:**

Rigaku Saturn724+ diffractometer	4043 independent reflections
Radiation source: fine-focus sealed tube	3601 reflections with *I* > 2σ(*I*)
Graphite monochromator	*R*_int_ = 0.022
ω scans	θ_max_ = 25.4°, θ_min_ = 3.0°
Absorption correction: multi-scan (*CrystalClear*; Rigaku, 2008)	*h* = −11→9
*T*_min_ = 0.728, *T*_max_ = 1.000	*k* = −12→11
10036 measured reflections	*l* = −19→28

### Refinement

**Table:**

Refinement on *F*^2^	Primary atom site location: structure-invariant direct methods
Least-squares matrix: full	Secondary atom site location: difference Fourier map
*R*[*F*^2^ > 2σ(*F*^2^)] = 0.030	Hydrogen site location: inferred from neighbouring sites
*wR*(*F*^2^) = 0.066	H-atom parameters constrained
*S* = 1.04	*w* = 1/[σ^2^(*F*_o_^2^) + (0.0344*P*)^2^ + 0.2887*P*] where *P* = (*F*_o_^2^ + 2*F*_c_^2^)/3
4043 reflections	(Δ/σ)_max_ = 0.002
263 parameters	Δρ_max_ = 0.68 e Å^−^^3^
0 restraints	Δρ_min_ = −0.38 e Å^−^^3^

### Special details

**Table:**

Geometry. All e.s.d.'s (except the e.s.d. in the dihedral angle between two l.s. planes) are estimated using the full covariance matrix. The cell e.s.d.'s are taken into account individually in the estimation of e.s.d.'s in distances, angles and torsion angles; correlations between e.s.d.'s in cell parameters are only used when they are defined by crystal symmetry. An approximate (isotropic) treatment of cell e.s.d.'s is used for estimating e.s.d.'s involving l.s. planes.
Refinement. Refinement of *F*^2^ against ALL reflections. The weighted *R*-factor *wR* and goodness of fit *S* are based on *F*^2^, conventional *R*-factors *R* are based on *F*, with *F* set to zero for negative *F*^2^. The threshold expression of *F*^2^ > σ(*F*^2^) is used only for calculating *R*-factors(gt) *etc*. and is not relevant to the choice of reflections for refinement. *R*-factors based on *F*^2^ are statistically about twice as large as those based on *F*, and *R*- factors based on ALL data will be even larger.

### Fractional atomic coordinates and isotropic or equivalent isotropic displacement parameters (Å^2^)

**Table:**

	*x*	*y*	*z*	*U*_iso_*/*U*_eq_	
Br1	0.45970 (3)	−0.41644 (2)	1.234842 (12)	0.03454 (9)	
Br2	1.07815 (3)	0.96352 (2)	0.907769 (15)	0.04347 (11)	
C1	0.3098 (3)	0.3035 (2)	0.92713 (11)	0.0229 (5)	
C2	0.5517 (3)	0.3754 (2)	0.96667 (11)	0.0217 (5)	
C3	0.5857 (3)	0.2828 (2)	1.01093 (11)	0.0249 (5)	
H3	0.6838	0.2748	1.0403	0.030*	
C4	0.4721 (3)	0.2021 (2)	1.01114 (11)	0.0224 (5)	
C5	0.5015 (3)	0.1044 (2)	1.05768 (11)	0.0250 (5)	
H5	0.6018	0.0889	1.0834	0.030*	
C6	0.3944 (3)	0.0364 (2)	1.06569 (11)	0.0237 (5)	
H6	0.2947	0.0559	1.0407	0.028*	
C7	0.4157 (3)	−0.0662 (2)	1.10957 (11)	0.0224 (5)	
C8	0.2879 (3)	−0.1289 (2)	1.10968 (11)	0.0264 (5)	
H8	0.1922	−0.1009	1.0840	0.032*	
C9	0.2998 (3)	−0.2319 (2)	1.14709 (11)	0.0281 (6)	
H9	0.2135	−0.2736	1.1462	0.034*	
C10	0.4420 (3)	−0.2715 (2)	1.18561 (11)	0.0249 (5)	
C11	0.5712 (3)	−0.2086 (2)	1.18849 (11)	0.0264 (5)	
H11	0.6663	−0.2346	1.2157	0.032*	
C12	0.5576 (3)	−0.1069 (2)	1.15050 (11)	0.0249 (5)	
H12	0.6444	−0.0648	1.1522	0.030*	
C13	0.6671 (3)	0.4638 (2)	0.96501 (11)	0.0235 (5)	
H13	0.7624	0.4602	0.9967	0.028*	
C14	0.6451 (3)	0.5488 (2)	0.92140 (11)	0.0239 (5)	
H14	0.5499	0.5494	0.8896	0.029*	
C15	0.7548 (3)	0.6417 (2)	0.91800 (11)	0.0232 (5)	
C16	0.7173 (3)	0.7171 (2)	0.86599 (11)	0.0262 (5)	
H16	0.6253	0.7041	0.8333	0.031*	
C17	0.8135 (3)	0.8107 (2)	0.86184 (12)	0.0289 (6)	
H17	0.7866	0.8603	0.8269	0.035*	
C18	0.9497 (3)	0.8291 (2)	0.91031 (12)	0.0271 (6)	
C19	0.9919 (3)	0.7544 (2)	0.96202 (12)	0.0305 (6)	
H19	1.0854	0.7664	0.9940	0.037*	
C20	0.8949 (3)	0.6622 (2)	0.96584 (11)	0.0268 (5)	
H20	0.9232	0.6127	1.0009	0.032*	
C21	0.1228 (3)	0.4306 (2)	0.82034 (13)	0.0367 (7)	
H21A	0.2234	0.4381	0.8198	0.044*	
H21B	0.0528	0.4108	0.7791	0.044*	
C22	0.0793 (4)	0.5525 (3)	0.83894 (15)	0.0501 (8)	
H22A	0.1538	0.5757	0.8789	0.060*	
H22B	−0.0179	0.5434	0.8425	0.060*	
C23	0.0681 (4)	0.6585 (3)	0.79286 (17)	0.0578 (9)	
H23A	−0.0001	0.6318	0.7524	0.069*	
H23B	0.0241	0.7323	0.8037	0.069*	
C24	0.2172 (4)	0.6943 (3)	0.79049 (17)	0.0617 (9)	
H24A	0.2892	0.7113	0.8311	0.093*	
H24B	0.2043	0.7680	0.7656	0.093*	
H24C	0.2535	0.6263	0.7732	0.093*	
S1	0.12232 (7)	0.30281 (6)	0.87109 (3)	0.03603 (18)	
N1	0.4088 (2)	0.38747 (17)	0.92361 (9)	0.0227 (4)	
N2	0.3308 (2)	0.21138 (17)	0.96785 (9)	0.0227 (4)	

### Atomic displacement parameters (Å^2^)

**Table:**

	*U*^11^	*U*^22^	*U*^33^	*U*^12^	*U*^13^	*U*^23^
Br1	0.04852 (18)	0.02501 (14)	0.03030 (16)	−0.00198 (12)	0.01552 (13)	0.00624 (10)
Br2	0.03001 (16)	0.02807 (16)	0.0752 (2)	−0.00696 (12)	0.02359 (15)	0.00722 (13)
C1	0.0247 (12)	0.0208 (12)	0.0245 (13)	0.0002 (11)	0.0111 (10)	−0.0011 (10)
C2	0.0242 (13)	0.0178 (11)	0.0265 (13)	−0.0001 (10)	0.0138 (11)	−0.0024 (10)
C3	0.0227 (12)	0.0242 (12)	0.0271 (14)	0.0013 (11)	0.0089 (10)	0.0017 (10)
C4	0.0270 (13)	0.0185 (11)	0.0255 (13)	0.0015 (11)	0.0143 (11)	−0.0006 (10)
C5	0.0255 (13)	0.0234 (12)	0.0254 (14)	0.0029 (11)	0.0089 (11)	0.0038 (10)
C6	0.0273 (13)	0.0215 (12)	0.0233 (13)	0.0011 (11)	0.0110 (11)	−0.0012 (10)
C7	0.0293 (13)	0.0180 (11)	0.0220 (13)	−0.0006 (10)	0.0122 (11)	−0.0026 (9)
C8	0.0241 (13)	0.0278 (13)	0.0272 (14)	0.0002 (11)	0.0099 (11)	0.0014 (10)
C9	0.0302 (14)	0.0290 (13)	0.0300 (14)	−0.0056 (12)	0.0170 (12)	0.0002 (11)
C10	0.0370 (14)	0.0200 (12)	0.0214 (13)	−0.0017 (11)	0.0153 (11)	−0.0006 (10)
C11	0.0286 (13)	0.0246 (12)	0.0243 (13)	0.0043 (11)	0.0084 (11)	−0.0006 (10)
C12	0.0286 (13)	0.0221 (12)	0.0265 (14)	−0.0037 (11)	0.0134 (11)	−0.0010 (10)
C13	0.0217 (12)	0.0229 (12)	0.0271 (14)	−0.0032 (10)	0.0106 (11)	−0.0029 (10)
C14	0.0241 (12)	0.0237 (12)	0.0248 (13)	−0.0028 (11)	0.0104 (10)	−0.0031 (10)
C15	0.0268 (13)	0.0187 (12)	0.0274 (14)	−0.0029 (10)	0.0141 (11)	−0.0036 (10)
C16	0.0263 (13)	0.0235 (12)	0.0264 (14)	−0.0022 (11)	0.0078 (11)	0.0003 (10)
C17	0.0316 (14)	0.0237 (13)	0.0342 (15)	−0.0009 (11)	0.0159 (12)	0.0048 (10)
C18	0.0251 (13)	0.0185 (12)	0.0429 (16)	−0.0034 (11)	0.0189 (12)	0.0000 (11)
C19	0.0218 (12)	0.0313 (14)	0.0358 (16)	−0.0043 (12)	0.0084 (11)	−0.0048 (11)
C20	0.0265 (13)	0.0268 (13)	0.0261 (14)	−0.0011 (11)	0.0092 (11)	0.0031 (10)
C21	0.0338 (15)	0.0372 (15)	0.0325 (16)	−0.0050 (13)	0.0055 (12)	0.0049 (12)
C22	0.061 (2)	0.0488 (18)	0.054 (2)	0.0073 (16)	0.0371 (17)	0.0083 (15)
C23	0.073 (2)	0.0448 (18)	0.067 (2)	0.0212 (17)	0.0398 (19)	0.0228 (17)
C24	0.075 (2)	0.0504 (19)	0.073 (3)	0.0016 (18)	0.043 (2)	0.0210 (17)
S1	0.0271 (3)	0.0365 (4)	0.0372 (4)	−0.0085 (3)	0.0044 (3)	0.0112 (3)
N1	0.0244 (11)	0.0200 (10)	0.0244 (11)	−0.0030 (9)	0.0103 (9)	0.0004 (8)
N2	0.0252 (11)	0.0202 (10)	0.0244 (11)	−0.0023 (8)	0.0114 (9)	0.0020 (8)

### Geometric parameters (Å, º)

**Table:**

Br1—C10	1.901 (2)	C13—H13	0.9300
Br2—C18	1.901 (2)	C14—C15	1.466 (3)
C1—N1	1.328 (3)	C14—H14	0.9300
C1—N2	1.334 (3)	C15—C16	1.395 (3)
C1—S1	1.770 (2)	C15—C20	1.397 (3)
C2—N1	1.358 (3)	C16—C17	1.385 (3)
C2—C3	1.382 (3)	C16—H16	0.9300
C2—C13	1.462 (3)	C17—C18	1.376 (3)
C3—C4	1.386 (3)	C17—H17	0.9300
C3—H3	0.9300	C18—C19	1.383 (3)
C4—N2	1.347 (3)	C19—C20	1.376 (3)
C4—C5	1.461 (3)	C19—H19	0.9300
C5—C6	1.326 (3)	C20—H20	0.9300
C5—H5	0.9300	C21—C22	1.479 (4)
C6—C7	1.466 (3)	C21—S1	1.816 (3)
C6—H6	0.9300	C21—H21A	0.9700
C7—C8	1.393 (3)	C21—H21B	0.9700
C7—C12	1.396 (3)	C22—C23	1.544 (4)
C8—C9	1.386 (3)	C22—H22A	0.9700
C8—H8	0.9300	C22—H22B	0.9700
C9—C10	1.377 (3)	C23—C24	1.497 (4)
C9—H9	0.9300	C23—H23A	0.9700
C10—C11	1.383 (3)	C23—H23B	0.9700
C11—C12	1.381 (3)	C24—H24A	0.9600
C11—H11	0.9300	C24—H24B	0.9600
C12—H12	0.9300	C24—H24C	0.9600
C13—C14	1.327 (3)		
			
N1—C1—N2	128.9 (2)	C20—C15—C14	122.8 (2)
N1—C1—S1	119.68 (17)	C17—C16—C15	121.7 (2)
N2—C1—S1	111.38 (17)	C17—C16—H16	119.2
N1—C2—C3	120.7 (2)	C15—C16—H16	119.2
N1—C2—C13	118.0 (2)	C18—C17—C16	118.9 (2)
C3—C2—C13	121.3 (2)	C18—C17—H17	120.6
C2—C3—C4	119.3 (2)	C16—C17—H17	120.6
C2—C3—H3	120.3	C17—C18—C19	121.0 (2)
C4—C3—H3	120.3	C17—C18—Br2	119.62 (19)
N2—C4—C3	120.6 (2)	C19—C18—Br2	119.33 (19)
N2—C4—C5	118.0 (2)	C20—C19—C18	119.6 (2)
C3—C4—C5	121.4 (2)	C20—C19—H19	120.2
C6—C5—C4	124.3 (2)	C18—C19—H19	120.2
C6—C5—H5	117.9	C19—C20—C15	121.1 (2)
C4—C5—H5	117.9	C19—C20—H20	119.4
C5—C6—C7	127.2 (2)	C15—C20—H20	119.4
C5—C6—H6	116.4	C22—C21—S1	112.6 (2)
C7—C6—H6	116.4	C22—C21—H21A	109.1
C8—C7—C12	117.9 (2)	S1—C21—H21A	109.1
C8—C7—C6	118.5 (2)	C22—C21—H21B	109.1
C12—C7—C6	123.7 (2)	S1—C21—H21B	109.1
C9—C8—C7	121.6 (2)	H21A—C21—H21B	107.8
C9—C8—H8	119.2	C21—C22—C23	112.3 (3)
C7—C8—H8	119.2	C21—C22—H22A	109.2
C10—C9—C8	118.7 (2)	C23—C22—H22A	109.2
C10—C9—H9	120.6	C21—C22—H22B	109.2
C8—C9—H9	120.6	C23—C22—H22B	109.2
C9—C10—C11	121.3 (2)	H22A—C22—H22B	107.9
C9—C10—Br1	118.99 (18)	C24—C23—C22	113.9 (3)
C11—C10—Br1	119.76 (18)	C24—C23—H23A	108.8
C12—C11—C10	119.3 (2)	C22—C23—H23A	108.8
C12—C11—H11	120.3	C24—C23—H23B	108.8
C10—C11—H11	120.3	C22—C23—H23B	108.8
C11—C12—C7	121.1 (2)	H23A—C23—H23B	107.7
C11—C12—H12	119.5	C23—C24—H24A	109.5
C7—C12—H12	119.5	C23—C24—H24B	109.5
C14—C13—C2	124.4 (2)	H24A—C24—H24B	109.5
C14—C13—H13	117.8	C23—C24—H24C	109.5
C2—C13—H13	117.8	H24A—C24—H24C	109.5
C13—C14—C15	127.0 (2)	H24B—C24—H24C	109.5
C13—C14—H14	116.5	C1—S1—C21	103.43 (12)
C15—C14—H14	116.5	C1—N1—C2	115.05 (19)
C16—C15—C20	117.7 (2)	C1—N2—C4	115.4 (2)
C16—C15—C14	119.4 (2)		
			
N1—C2—C3—C4	−0.1 (3)	C20—C15—C16—C17	−1.1 (4)
C13—C2—C3—C4	179.2 (2)	C14—C15—C16—C17	176.6 (2)
C2—C3—C4—N2	1.6 (3)	C15—C16—C17—C18	0.2 (4)
C2—C3—C4—C5	−178.7 (2)	C16—C17—C18—C19	1.2 (4)
N2—C4—C5—C6	−10.4 (3)	C16—C17—C18—Br2	−176.17 (18)
C3—C4—C5—C6	170.0 (2)	C17—C18—C19—C20	−1.6 (4)
C4—C5—C6—C7	177.3 (2)	Br2—C18—C19—C20	175.71 (18)
C5—C6—C7—C8	−178.2 (2)	C18—C19—C20—C15	0.7 (4)
C5—C6—C7—C12	0.3 (4)	C16—C15—C20—C19	0.6 (4)
C12—C7—C8—C9	−2.8 (3)	C14—C15—C20—C19	−177.0 (2)
C6—C7—C8—C9	175.8 (2)	S1—C21—C22—C23	−175.6 (2)
C7—C8—C9—C10	1.0 (4)	C21—C22—C23—C24	−67.4 (4)
C8—C9—C10—C11	1.6 (4)	N1—C1—S1—C21	0.2 (2)
C8—C9—C10—Br1	−177.69 (18)	N2—C1—S1—C21	−178.25 (17)
C9—C10—C11—C12	−2.2 (3)	C22—C21—S1—C1	−90.2 (2)
Br1—C10—C11—C12	177.02 (18)	N2—C1—N1—C2	1.8 (3)
C10—C11—C12—C7	0.3 (3)	S1—C1—N1—C2	−176.31 (16)
C8—C7—C12—C11	2.1 (3)	C3—C2—N1—C1	−1.4 (3)
C6—C7—C12—C11	−176.4 (2)	C13—C2—N1—C1	179.2 (2)
N1—C2—C13—C14	−5.7 (3)	N1—C1—N2—C4	−0.4 (3)
C3—C2—C13—C14	174.9 (2)	S1—C1—N2—C4	177.84 (16)
C2—C13—C14—C15	178.4 (2)	C3—C4—N2—C1	−1.4 (3)
C13—C14—C15—C16	174.3 (2)	C5—C4—N2—C1	179.0 (2)
C13—C14—C15—C20	−8.1 (4)		
