# Supplementary material for: Crystal structure of 4,6-bis­[(E)-4-bromo­styr­yl]-2-(butyl­sulfan­yl)pyrimidine
Source: Acta Crystallogr Sect E Struct Rep Online. 2014 Nov 21;70(Pt 12):o1282. doi: 10.1107/S1600536814024714 (PMC4257452; doi:10.1107/S1600536814024714)
Supplement: Supplementary file 4 [file e-70-o1282-Isup4.doc]

**Crystal structures of 4,6-bis((E)-4-bromostyryl)-2-(butylthio)pyrimidine**

**Wang Yu, Jingbao Song and Aijian Wang**

**S1. Comment**

Pyrimidine, as a widespread heterocycle molecule, plays a vital role in chemistry and molecular biology owing to its enormous chemical, biological and pharmaceutical properties (Walker *et al.*, 2009; Laar et al., 2001; Deng et al., 2008; Nguyen, 2008). Pyrimidine derivatives are often used as organic ligand in crystal engineering and supramolecular chemistry. In this paper we report the structure of a bis-styryl pyrimidine derivative, 4,6-bis((E)-4-bromostyryl)-2-(butylthio)pyrimidine.

As shown in Figure 1. The pyrimidine ring makes dihedral angel of 11.02 (11)° and 13.20 (12)° with respect to both plane generated through the atoms of the benzene ring 2 (C7-C12) and benzene ring 3 (C15-C20). The bond length of Br1-C10 is 1.901 (2) Å. The bond length of C4-C5 and C6-C7 are 1.461 (3) and 1.466 (3) Å, respectively, which are obviously shorter than the bond length of C22-C23 (1.544 (4) Å). Hence, the shorter bond length indicates a character between the single and double bond, which demonstrates a considerable electron delocalization of the conjugate molecule. The weak molecular interaction is the main factor that influences the molecule arrangement and crystal packing. As shown in Figure 2, the interaction distance of C-H···π interaction between C22 and its neighbor benzene ring Cg3 is 3.8206 (41) Å. Furthermore, π-π stacking interaction is found in its crystal packing, and the pyrimidine ring stacks with its nearby molecule with angel offset and distance of 11.02 (11)° and 3.6718 (8) Å, respectively. The C-Br···π interaction is formed between C10-Br1 and benzene ring 2 (C7-C12). The interaction distance is 3.7025 (7) Å.

**S2. Experimental**

The product was synthesized following a similar procedure as described elsewhere (Liu *et al.*, 2007). 2-(Butylthio)-4,6-dimethylpyrimidine (2.95 g, 15 mmol) and bromobenzaldehyde (6.1 g, 33 mmol) were added in an aqueous solution of sodium hydroxide (5 M, 50 ml) containing tetrabutylammonium iodide (10 mol % versus the heterocycle) and mixed. The mixture was heated under reflux for 3 h. After cooling, the reaction mixture was extracted with dichloromethane (120 ml × 3). The extract solution was dried with magnesium sulfate. After removal of the drying agent by filtration, the solvent was removed by evaporation under reduced pressure. The crude product was recrystallized to afford the desired pale-yellow micro-crystals (5.17 g, yield 65%). ^1^H NMR: (400 MHz, CDCl_3_), δ (ppm): 1.027 (t, J =7.2 Hz, 3H), 1.525-1.617 (m, 2H), 1.798-1.872 (m, 2H), 3.275 (t, J = 7.2 Hz, 2H), 6.918 (s, 1H), 6.994 (d, J = 15.6 Hz, 2H), 7.466 (d, J = 8.4 Hz, 4H), 7.540-7.820 (dd, J = 7.2, 1.6 Hz, 4H), 7.840 (d, J = 16 Hz, 2H).

**S3. Refinement**

All H atoms were geometrically placed and included in the refinement in the riding-model approximation, with isotropic displacement parameters set to *U*_iso_(H) = 1.5*U*_eq_(C) for methyl H atoms and 1.2*U*_eq_(C) for other.


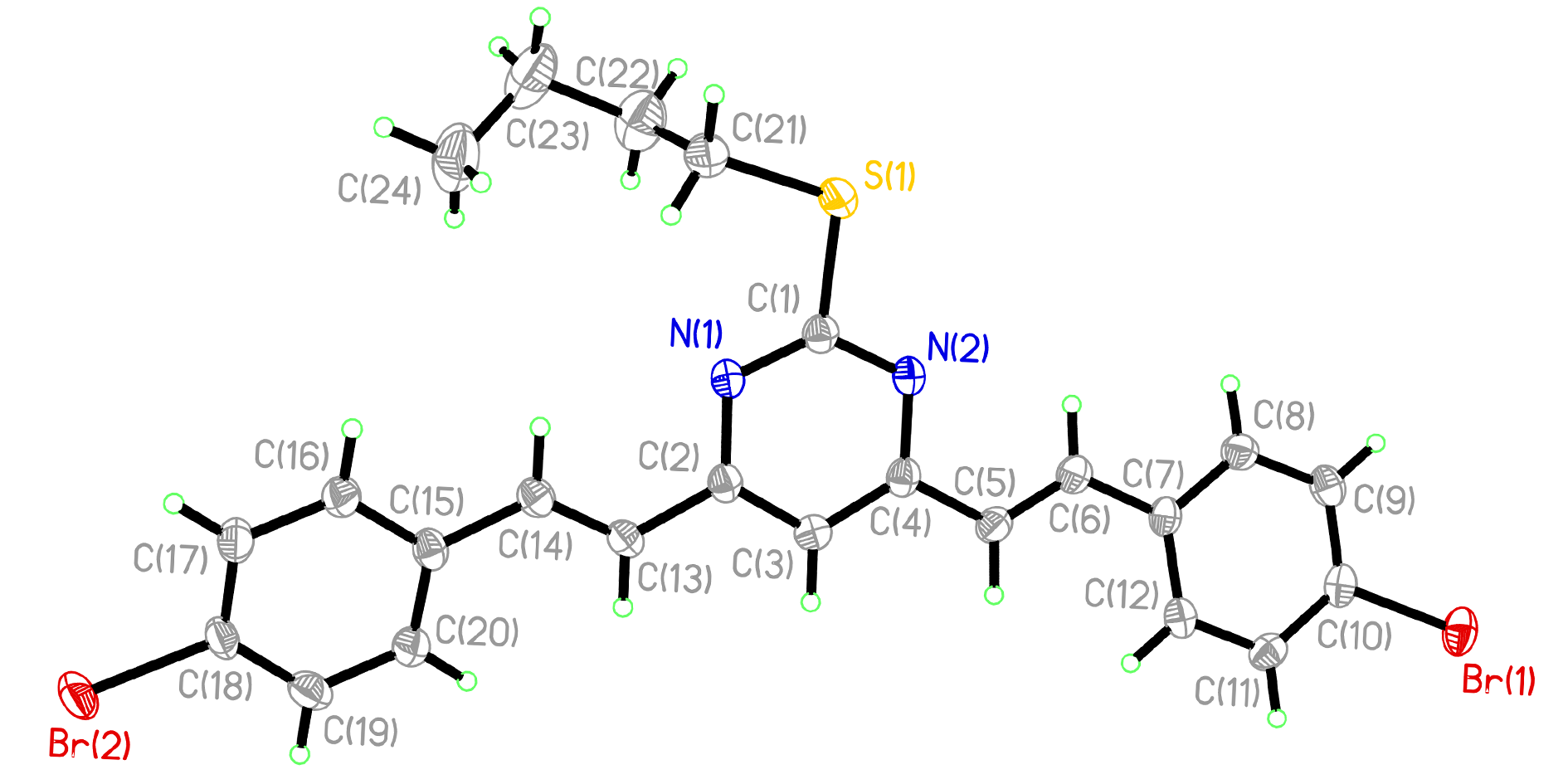


**Figure 1**

View of the title compound with displacement ellipsoids drawn at the 50% probability level.


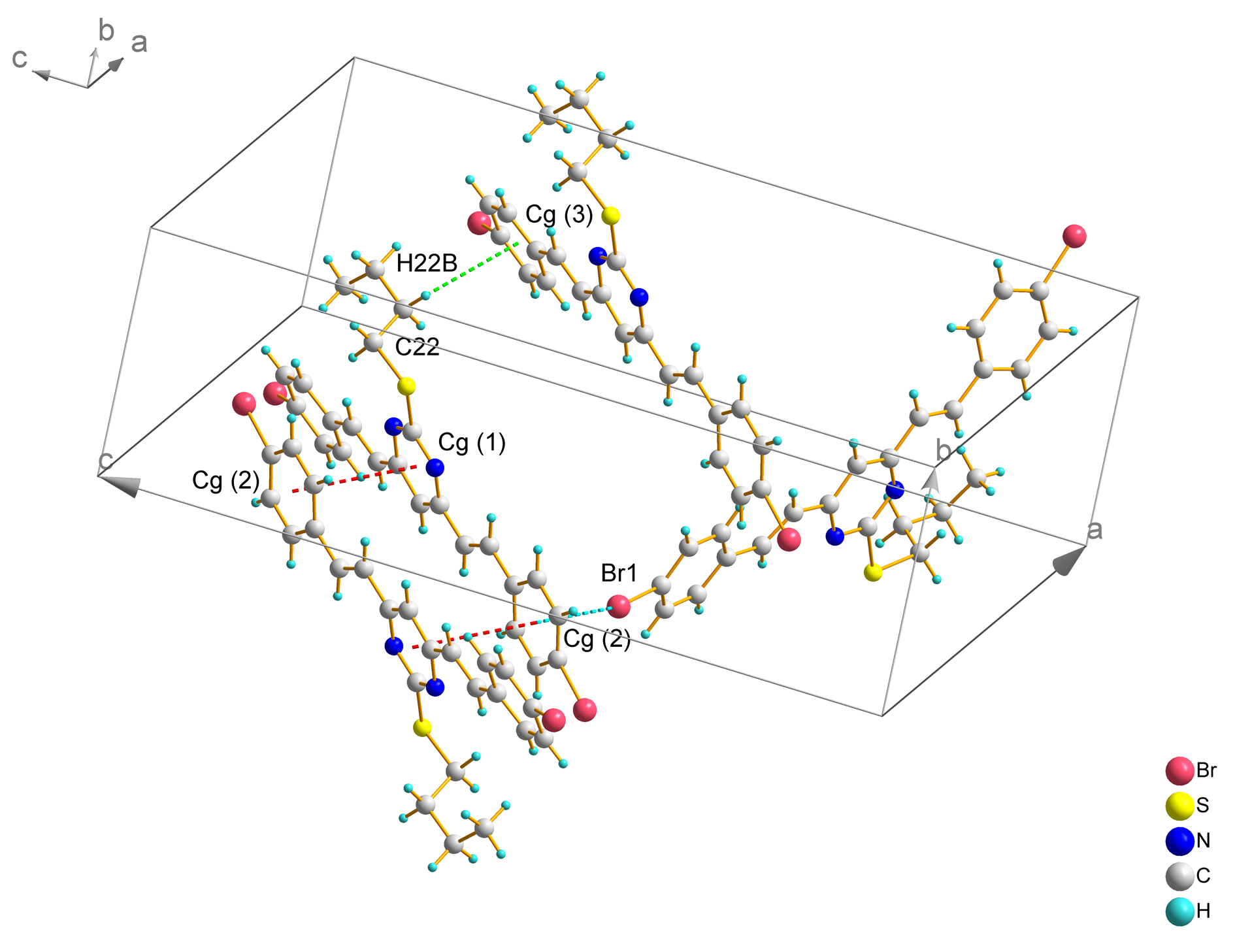


**Figure 2**

The intermolecular interaction in molecule structure of the title compound, Cg denotes the relevant ring centroid.
