# Supplementary figures and images for: Crystal structure of 4,6-bis­[(E)-4-bromo­styr­yl]-2-(butyl­sulfan­yl)pyrimidine
Source: Acta Crystallogr Sect E Struct Rep Online. 2014 Nov 21;70(Pt 12):o1282. doi: 10.1107/S1600536814024714 (PMC4257452; doi:10.1107/S1600536814024714)

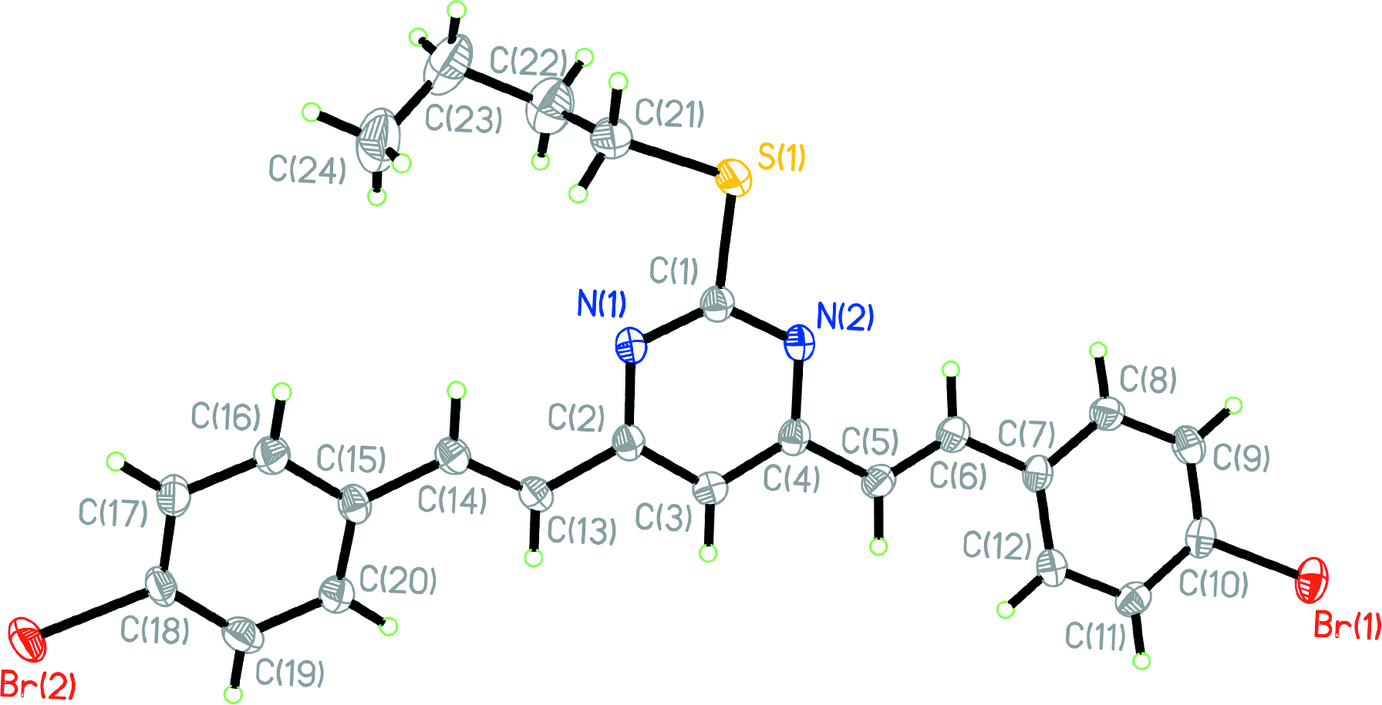

Supplement: Supplementary file 5 [file e-70-o1282-fig1.tif]

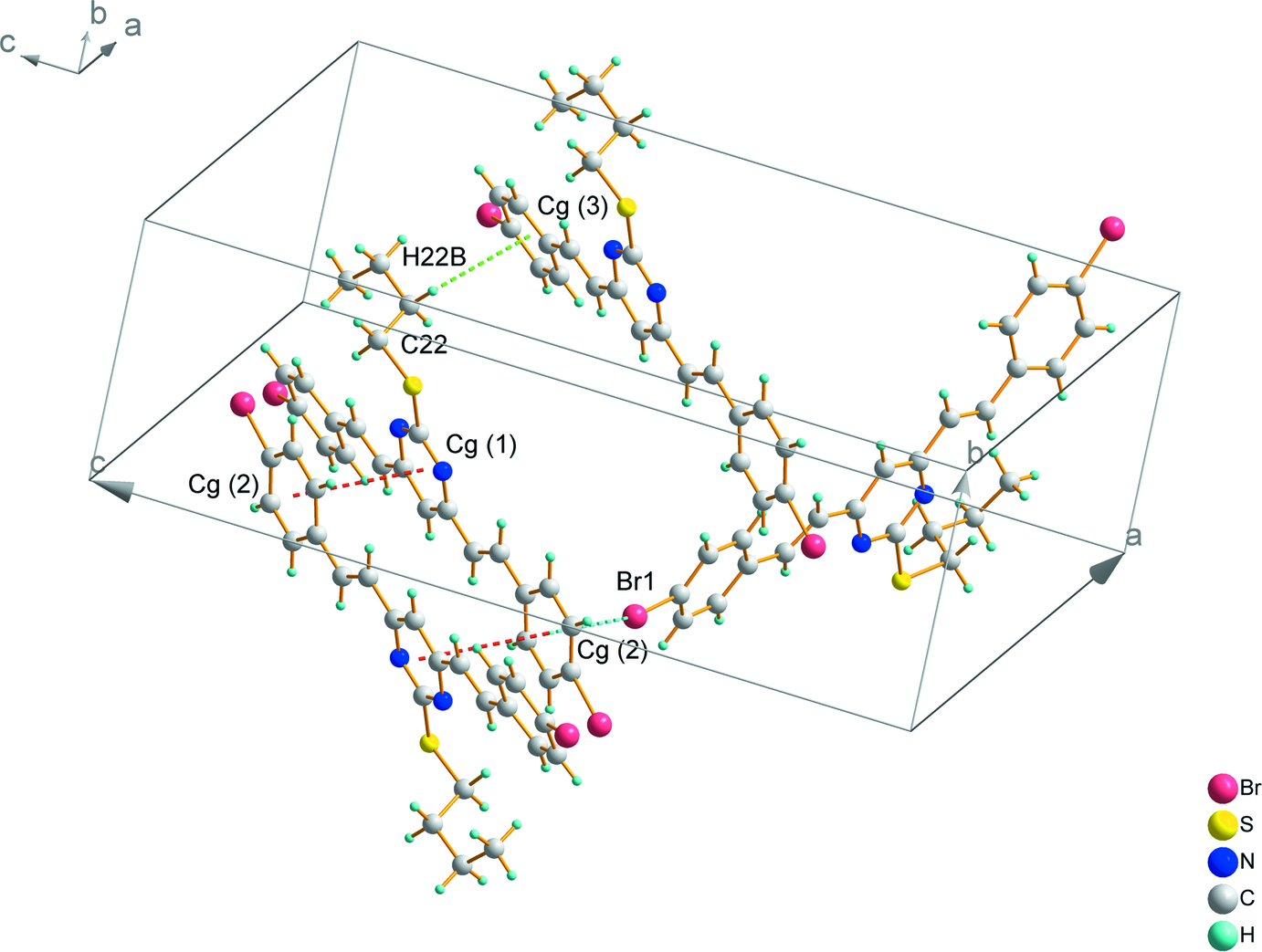

Supplement: Supplementary file 6 [file e-70-o1282-fig2.tif]
